# Nanochitosan: Commemorating the Metamorphosis of an ExoSkeletal Waste to a Versatile Nutraceutical

**DOI:** 10.3390/nano11030821

**Published:** 2021-03-23

**Authors:** Iyyakkannu Sivanesan, Manikandan Muthu, Judy Gopal, Nazim Hasan, Syed Kashif Ali, Juhyun Shin, Jae-Wook Oh

**Affiliations:** 1 Department of Bioresources and Food Science, Konkuk University, 120 Neungdong-ro, Gwangjin-gu, Seoul 05029, Korea; isivanesan@gmail.com; 2 Laboratory of Neo Natural Farming, Chunnampet, Tamil Nadu 603 401, India; bhagatmani@gmail.com (M.M.); jejudy777@gmail.com (J.G.); 3 Department of Chemistry, Faculty of Science, Jazan University, Jazan P.O. Box 114, Saudi Arabia; hhasan@jazanu.edu.sa (N.H.); skali_169@yahoo.com (S.K.A.); 4 Department of Stem Cell and Regenerative Biotechnology, Konkuk University, Seoul 05029, Korea; junejhs@konkuk.ac.kr

**Keywords:** chitin, chitosan, nanochitosan, drug delivery, biomedical applications, nutraceutical

## Abstract

Chitin (poly-*N*-acetyl-D-glucosamine) is the second (after cellulose) most abundant organic polymer. In its deacetylated form—chitosan—becomes a very interesting material for medical use. The chitosan nano-structures whose preparation is described in this article shows unique biomedical value. The preparation of nanochitosan, as well as the most vital biomedical applications (antitumor, drug delivery and other medical uses), have been discussed in this review. The challenges confronting the progress of nanochitosan from benchtop to bedside clinical settings have been evaluated. The need for inclusion of nano aspects into chitosan research, with improvisation from nanotechnological inputs has been prescribed for breaking down the limitations. Future perspectives of nanochitosan and the challenges facing nanochitosan applications and the areas needing research focus have been highlighted.

## 1. Introduction

Henri Braconnot, in 1811, discovered chitin in mushrooms. Rouget in 1859, heated chitin in an alkaline medium to get an organic acid-soluble material [1] and Hoppe Seyler (1894) named this acid-soluble material as chitosan. In 1950 the structure of chitosan was elucidated. Crini [2] has extensively reviewed the 220 years of chitin history. Chitosan (CS) is obtained from chitin. Chitin is naturally extracted from yeast, fungi, algae, silkworms, cockroaches, honey bees and marine aquatic animals such as arthropods, crustaceans, cephalopods, lobsters, mollusks and shrimps [3]. *α*-Chitin in the 3D form is also reported to be found in sponges [4]. CS is obtained from the deacetylation of chitin. It is a linear and cationic biodegradable carbohydrate polymer. It is non-toxic and renewable [5]. CS and chitin are differentiated on the basis of the N-acetylamine group. Chitin has an amino group at the C2 position, whereas chitosan is a product of alkaline or enzymatic deacetylation of chitin. The degree of deacetylation (DD) of chitin ranges from 60% to 100% [6]. It consists of randomly distributed *β*-(1-4)-linked *N*-acetyl-D-glucosamine (GlcNAc) and D-glucosamine (GlcN). It is the occurrence of the amino group at the C2 position, that result in the superior properties of chitosan [6,7,8]. These superior properties of CS include anticoagulant properties, fluidity, water solubility and high water reducing ratio [9,10] and biological properties such as controlled drug delivery, gelation, enhancement of permeation, mucoadhesion, targeting the colon and efflux pump inhibition [11,12,13]. CS is a high-value biomacromolecule, owing to its use in biomedical applications. Biocompatibility and low toxicity, immunity, and biodegradability are the key traits that have made CS biomedically significant. The antimicrobial property of chitosan has been reported to be high at low pH because of the cationic amino group [14]. The adhesive nature of chitosan enables its tissue adherence, extending its leverage in dentistry, orthopaedics, wound healing and ophthalmology [15,16,17]. CS also possesses a high affinity for negatively charged cell membranes, thus showing enhanced site-specific targeting abilities [18]. 

Inspite of the unique properties of CS, its clinical applications are limited by its poor mechanical properties [19,20]. Mechanical properties such as, modulus of elasticity, tensile strength, elongation, hardness and fatigue limit, which are essentials for any biomaterial, are those that at a deficit in CS. Hence, to overcome these limitations, CS has been subjected to a number of modifications [21,22]. Free amino and hydroxyl groups have generated various other CS derivatives [23,24], that have been applied for biomedical and pharmaceutical processes, such as tissue engineering, drug delivery, gene delivery, vaccine delivery, wound healing and cosmetics [25,26,27]. 

**Figure 1:**
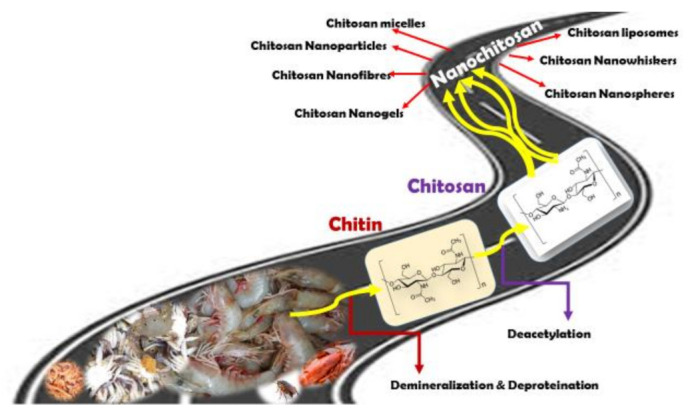
Tracing the journey from shell waste to nanochitosan.

What makes chitosan attractive, is the fact that it is derived from chitin and that chitin is obtained from the exoskeleton of various classes of organisms mentioned above. With the source material being shrimp, squid, lobster, crab shell wastes, this valuable asset is an added asset. This makes this material a cost-effective and renewable resource extracted from crustacean waste generated from the seafood industry. The general process of isolation of chitin from natural sources is through demineralization and deproteinization of the waste material with strong acids and bases, such as HCl and NaOH [28]. Through enzymatic or chemical deacetylation, chitin can be converted to its most well-known derivative, CS. The various methods involved in the isolation of chitin and recovery of chitosan from chitin have been well researched and reviewed [29,30,31]. Figure 1 gives the overall scheme of the journey from shell wastes to nanochitosan.

In this current review, the preparation of nano chitosan-based materials from chitin and chitosan has been briefly presented. The unique enhanced biomedical applications through nano-aturization of chitosan have been summarized and the future perspectives presented. 

## 2. Preparation of Nanochitosan

Over the last few decades, novel nanomaterials have been successfully developed from CS and widely applied for targeted drug delivery. CS is well-known for drug delivery based on the following features: (1) biocompatibility, (2) defending drug molecules from gastric acids and blood flow responses, (3 adherence to mucosal tissues to improve drug absorption, (4) binding with anionic DNA by electrostatic action and (5) targeted colon administration [32,33,34]. Reviews on chitosan-based nanoparticle systems for disease treatment have been published reporting the biological applications of CS [35,36]. Moreover, since the CS backbone has multiple free amino and hydroxyl groups, these have been used for the construction of CS-based nanomaterials, such as nanogels, nanoparticles (NPs), micelles, liposomes, nanofibers, and nanospheres. These CS-based nanomaterials have been used for various biomedical applications [37,38,39]. Materials reported to be used during the preparation of CS nanoparticles/CS composites include, polymers such as dextran sulfate [40], sodium alginate [41,42], carrageenan [43], arabic gum [44], glucomannan [45], carboxymethyl cellulose [46], chondroitin sulfate [47], pectin [48], heparin [49], hyaluronic acid [50], sodium lauryl sulfate [51], cyclodextrins [52], poly-*γ*-glutamic acid [53,54] and poly(acrylic acid) [55], insulin [56] and occasionally DNA [57,58]. Examples of such composites are chitosan/alginate/tripolyphosphate [59], chitosan/glucomannan/tripolyphosphate [60], chitosan/hyaluronic acid/tripolyphosphate [50] or chitosan/cyclodextrin/tripolyphosphate [52] nanoparticles.

### Methods of Nanochitosan Preparations

Ever since the first report in 1994 by Ohya et al. [61], diverse techniques have been optimized for the preparation of CSNPs. Methods such as emulsification and varied kinds of coacervation and their respective modifications. Emulsion-droplet coalescence [62], emulsion solvent diffusion [63], reverse micellar method [64], ionic gelation, polyelectrolyte complexation [41,65] and desolvation [66] are some of the prevalent methodologies that are in practice. These generally are prototypes of bottom-up fabrication processes, comprising of assembly of molecules to yield defined nanostructures [67] which display size polydispersity [68]. It is assumed that larger nanoparticles possess higher drug loading capacity, while smaller nanoparticles have the advantage of being able to easily assess tissues or cells. Hence, given this fact either sizes stand their own advantage.

Ionic gelation involves, dissolving CS in an aqueous acidic solution to obtain cationic chitosan [69]. This solution is added to anionic tripolyphosphates (TPP) solution to yield spherical particles [70,71]. In an emulsion cross-linking method, the aqueous CS solution is emulsified in the oil phase and the aqueous droplets are stabilized using a suitable surfactant,. Then, glutaraldehyde (cross-linking agent) is added to precipitate nanoparticles [61,72,73]. Spray-drying is another method where, CSNPs are prepared by use of a nano-spray dryer [74,75,76,77]. Emulsion droplet coalescence is widely reported for drug delivery applications, a stable emulsion containing an aqueous solution of CS with the drug is introduced into liquid paraffin oil. CS aqueous solution in NaOH in liquid paraffin oil is another emulsion system, these different emulsions are mixed under high-speed stirring, to precipitate CS droplets to obtain small size particles [78,79]. The same group applied the above technology to prepare gadolinium-loaded chitosan nanoparticles, for neutron-capture therapy of cancer [78]. 

The emulsion solvent diffusion method is an adaptation of the procedure that produces PLGA-based nanoparticles [80]. This involves the addition of an organic phase to an aqueous solution containing CS and a stabilizer [63]. Nanoprecipitation is another technique, where CS is dissolved in a suitable solvent to form the diffusing phase, which is added to the dispersing phase with small amounts of polysorbate-80 to yield smaller NPs [81]. Reverse micellisation, [82,83], uses a lipophilic surfactant (sodium bis (ethyl hexyl) sulfosuccinate or acetyl trimethyl ammonium bromide) dissolved in n-hexane. CS solution, drug and glutaraldehyde are added to the organic phase under continuous stirring to obtain nanoparticles [64,84,85]. Desolvation/simple coacervation/phase separation, is another process reported for obtaining nanochitosan particles [86,87,88]. Modified ionic gelation uses aqueous acrylic acid monomer solution in aqueous CS solution for ionic gelation [55,89,90]. Emulsion solvent diffusion [63], ionic gelation and polyelectrolyte complexation methods [91,92] have also been reported. Grenha, 2012 [93] have elaborated extensively on the preparation techniques and the ensuing chitosan nanoparticle applications in their excellent review.

## 3. Biomedical Milestones of Nanochitosan

Nanomedicine has led to ennumerable breakthroughs in the detection, diagnosis, and treatment of various diseases [94]. Nanochitosan have been proven as drug carriers, for controlled drug release. Chitosan has been able to improve drug solubility and stability, enhance efficacy and reduce toxicity.

### 3.1. Antitumor Applications of Nanochitosan

The antitumor effects of chitosan, confirm their prospective application as an antitumor drug and as a drug carrier [95]. Due to unique features such as compatibility and biodegradability, nanochitosan have emerged as a vital tool for drug delivery applications specifically for cancer [96]. Nanochitosan have been deployed for the delivery of anti-cancer drugs like methotrexate [97], epirubicin [98], curcumin [99,100], 5-flourouracil [101,102], cysplatin [103], mitomycin C [104], paclitaxel [105], and tamoxifen [106], docetaxel [107], doxorubicin (DOX) [64,91]. Nanochitosan has been successful in focusing the anti-tumor efficacy, control release and by drug targeting towards tumor (reducing widespread toxicity). Nanochitosan have been successful in, releasing 50% of methotrexate loaded in 48 h [97], releasing the drug cysplatin slowly [103], loading epirubicin into cholesterol-modified CS, followed by their pH-dependent release in vitro [98], encapsulation and release of doxorubicin in CS-based NPs [91], encapsulation of mitomycin C (chemotherapeutic) for bladder cancer cells therapy [104], reducing drug toxicity and tumor volume in mice [107], carrier/vehicle/prodrug of cancer therapy [100,105,108,109,110], encapsulation of dextran-doxorubicin conjugate in CSNPs [64], a controlled release increase tamoxifen chemotherapeutic efficiency [106] and chemotherapy of breast cancer to reduce traditional chemo-related side-effects [102].

### 3.2. Drug Delivery Applications of Nanochitosan

Nano mucosal delivery carriers are useful for mucosal drug delivery because they are prone to obstruction and have need protection [111]. Due to its mucoadhesive property, nanochitosan has been extensively used for this purpose [112,113,114]. De Campos et al. have shown that CS nanoparticles remained attached to the rabbits’ cornea and conjunctiva for up to 24 h [115]. Other studies have confirmed the use of mucoadhesive chitosan (CS)-sodium Alg nanoparticles for prolonged topical ophthalmic delivery of the antibiotic, gatifloxacin [116,117]. Sarah Baltzley et al. have reported the use of nanochitosan particles for intranasal delivery in order to amplify olanzapine systemic bioavailability [118]. Abeer M. Al-Ghananeem et al. reported nanoCS for intranasal delivery for didanosine systemic and brain targeting [119]. Park et al. demonstrated targeted delivery to the liver using galactosylated-chitosan–graft-dextran DNA complexes [120]. Similarly, transferrin–chitosan–DNA nanoparticles have been employed for targeted drug delivery [93]. Nanochitosan have been reported for mucosal delivery of antigen vaccines [121,122,123,124,125,126,127,128,129,130,131,132]. Nanochitosan are suggested to be ideally useful for modern vaccinology, facilitating oral and nasal delivery of nanoparticles with protective immune responses. The nanosize dimensions of nanochitosan aids in their effective uptake by M cells, in mucosa, gut, nasal and bronchus-associated lymphoid tissues [122]. 

Nanochitosan have been widely reported for drug delivery with respect to infectious diseases. The functional groups aid in guiding the loaded drugs to the infection site. Sustained biodegradation of chitosan helps in the controlled and slow release of loaded moieties and is competent in reducing dosing frequency [133,134,135]. CS nanoparticles have been reported in delivery of anti-microbial drugs such as: cefazolin [136], rifampicin [137,138,139], isoniazid [138,140] and tetracycline [141], amphotericin B [142,143,144],vancomycin [145,146], daptomycin [147], ofloxacin [148], ciprofloxacin [140], amoxicillin [149]. Nanochitosan have been confirmed to exhibit better encapsulation efficiency (EE), good stability against *Klebselia pneumonia*, *P. aeruginosa* and lactamase positive *E. coli* [136]. Other properties of nanochitosan towards antimicrobicidal effects include, rapid bactericidal activity and reduced dose frequency [137], high entrapment efficiency, sustained release, prolong residence time [143], inhibition of *H. pylori* [149]. Bivas-Benita et al. [150] demonstrated the pulmonary delivery of DNA vaccines against tuberculosis. 

CS is also recognized for oral drug delivery [151]. Nanochitosan has been successfully used against ulcerative colitis, Crohn’s disease, pseudomembranous colitis and irritable bowel syndrome [152,153]. CS is recognized as the most predominant polymer with respect to colon targeted delivery. This is owing to the fact that it dissolves in acidic pH of the stomach and swells up at intestinal pH ranges [154]. Nanochitosan have been reported to improve uptake of HT-29 cell and colorectal cancer [155]. The release of 5-aminosalicylic acid from nanochitosan based on ion-exchange mechanism is reported [156]. S-DNA chitosan nanoparticles were more stable in the upper regions of the small intestine [157] and have been reported for targeting drugs to colon tumor [158] and have been also reported for endoscopic detection of colorectal cancer [159] and apoptosis initiation by trans-retinoic acid bearing methoxy poly(ethylene glycol)-grafted nanochitosan [160]. Moreover, hyaluronic acid coupled CSNPs were 60-fold more effective on HT-29 cells [161].

Nanochitosan have been extensively used for ocular drug delivery. Drug delivery of CyA [115,162], FITC-BSA [163,164], indomethacin [165,166], pilocarpine [167,168], pDNA [169], and prednisolone [170] has been successfully accomplished. The transdermal route is usually a more “patient-friendly” approach overcoming stomach-based side effect [171]. Raida Al-Kassas et al. used nanochitosan particles dispersed in mucoadhesive gel for transdermal delivery [172]. In another study, Anita Hafner et al. investigated the application of nanochitosan particles for transdermal melatonin [173].

CSNPs have been applied for anti-inflammatory drug delivery of drugs such as zaltoprofen [174], hydrocortisone [175], ketorolac tromethamine [176], tretinoin [177]. The use of nanochitosan particles has reported increasing anti-inflammatory activity and efficacy as well as physical stability. CSNPs have also been used for anti-HIV drugs such as, lamivudine [178], zidovudine [179]; anti-malarial drugs such as chloroquine [180], antitubercular drugs including rifampicin and isoniazid [138]; muscle relaxants like thiocolchicoside [181] and oral hypoglycemic medications such as insulin [182]. 

### 3.3. Miscellaneous Applications of Nanochitosan

CS-based DNA flu vaccine for intranasal administration has also been formulated and demonstrated [132,133]. Smitha et al. reported amidase encapsulated O carboxymethyl CSNPs against *S. aureus* infections. Nanochitosan has been established as the most suitable candidate for the oral vaccine delivery system [134]. Pattani et al. [135] studied the immunological and membrane effects of nanochitosan in the wound-healing process. CS–TPP nanoparticles have demonstrated a higher potential for safe and cost-effective delivery of siRNA [183,184]. Nanochitosan particles have been reported as non-viral vectors for gene delivery and carriers for protein molecules [185,186,187].

**Figure 2:**
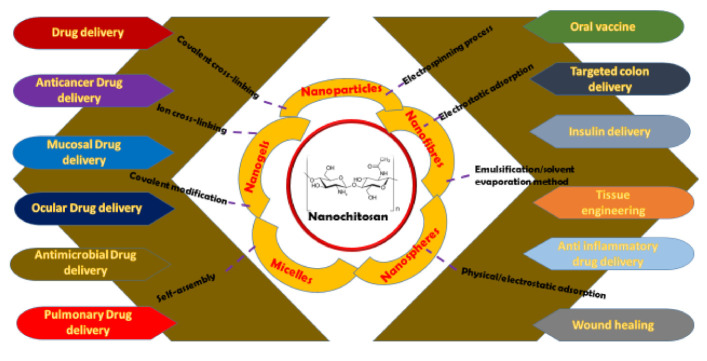
Overview of nanochitosan preparation techniques and applications.

CS is of high value when it comes to tissue engineering. Being a natural polymer, CS displays excellent biocompatibility [188]. With respect to insulin delivery, CS–dextran sulphate and chitosan Alg nanoparticles have been used as insulin and alternate polypeptides’ carriers [41]. Nanochitosan particles have been reported to enhance the systemic absorption of insulin upon nasal instillation [189]. Insulin-loaded nanochitosan particles have been effective in reducing glucose levels in a diabetic rat model [53]. Figure 2 gives a bird’s eye overview of Section 2 and Section 3.

## 4. Challenges Facing Nanochitosan Applications and Future Perspectives

CS by itself is a valuable asset, given the fact that it is recovered from exoskeletal wastes. CS recovery has brought meaningful utility to the tons of marine shell waste being dumped into the environment. Through this retrieval, dual purposes are solved through the proper usage of marine wastes for generating a versatile product. Nanochitosan has further stretched the limits of utility of this versatile product in leaps and bounds. As in every case, nano has certainly pushed the limits of bulk materials way beyond human perception, in case of CS too, as expected outcomes have been realized. As summarized in the earlier sections, nanochitosan has come a long way and nanomedicine has greatly benefitted from its inputs. 

### 4.1. Clinical Challenges of Nanochitosan

Discussing the challenges facing nanochitosan biomedical applications, it needs to be highlighted that the clinical administration of CS-based drug vectors still faces a limitation owing to their undisclosed risks. A nasal formulation of morphine (RylomineTM) based on CS is currently in Phase 2 clinical trials (UK and EU) and Phase 3 clinical trial. It is predicted that when it lands on the market it will encounter comparison with similar products and may be answerable for the unforeseen impacts on humans [190]. CS by itself has very few reports for their clinical studies or trials, nanochitosan based work is even more scarce. This apparently indicates that there is a gap between theory and practice, hence this needs to be worked on. Whatever may be the credentials of a nanomaterial for biomedical applications, if it does not progress from benchtop to bedsides, no progress is real progress. 

Moreover, in vivo studies involving nanochitosan are also merely a handful, in vivo studies are the preludes to clinical trials, these need to be initiated and the reasons why these haven’t been initiated need to be assessed and addressed.

### 4.2. Limiting Challenges in Nanomodifications of Chitosan

Few nanomodifications of chitosan have been reported (with nanochitosan particles being the most predominant), when there are numerous nano morphologies and unique properties specific to each of these morphologies, varied synthesis methods need to be worked on and tested for their biomedical applications. A distinct list as to the limitation of nanochitosan with respect to biomedical applications is yet to emerge. Only when we know the disclosure of the limitations we have hit on, will there be possibilities to probe overcoming those.

Composites in materials science, have enabled considerable advances and breakthroughs, since properties of two or three different materials combine and their strengths integrate and their weaknesses overcome. This review has presented the handful of chitosan composites reported thus far, Table 1 gives the consolidated list of the chitosan related composites and their inputs in biomedical applications [190,191,192,193,194,195,196,197,198,199,200,201,202,203,204,205,206,207,208,209,210,211,212,213,214,215,216,217,218,219,220,221,222,223,224,225,226,227]. As observed from the table, there are not many chitosan composites, the diversity of the composites are also limited, most of the composites involve chitosan and other polymers and acid moieties. The introduction of composites has been essentially useful in extending the application of CS to rather demanding biomedical aspects such as, wound dressing, antimicrobial, skin tissue engineering, bone grafting, wound healing and the like. Nanochitosan composites involving metal nanomaterials is the least (carboxymethyl chitosan-PVA/Ag nanoparticles and gold nanocluster-conjugated chitosan) represented, also composites mostly involve chitosan associations with other polymers, while nanochitosan based composites are meagerly represented. Few studies involving chitosan nanofiber and nanoparticle based composites are those that are available, there is definitely a lot more to explore and improvisations to be made through the integration of materials.

### 4.3. Limitations in Biomedical Applications of Nanochitosan 

As much as drug delivery of nanochitosan has been worked on, not much has been done with respect to other biomedical applications. Biomedical aspects such as, tissue engineering, wound healing, antimicrobial (especially antiviral) activity, are all areas craving attention. Much of the research focus of nanochitosan has been targeted in and around drug delivery applications, leading to narrowing down the scope of biomedical applications of nanochitosan to this area alone. This review found reasonable lack of reports in this direction, which we highlight as concerns that could eventually become directions for future perspectives.

## 5. Conclusions

The credentials of shell waste-derived nanochitosan have been reviewed and the lacunae in taking this cost-effective and renewable resource towards clinical applications are speculated and discussed. The preparation of nanochitosan and the biomedical applications of nanochitosan have been reviewed. The limitations and challenges facing the expansion and deployment of nanochitosan to clinical settings has been addressed. The very reason that nanochitosan has been retrieved from exoskeletal wastes, makes it a valuable asset and this review emphasizes that there is more future and promises to lie ahead inspite of the challenges.

## Figures and Tables

**Table 1:** Examples of nanochitosan composites applied in biomedical applications.

Nanochitosan composites	Biomedical Application	Associated Drug	Reference
Chitosan + alginate + Pluronic	Drug delivery	Curcumin	[191]
Poly(*β*-malic acid)-γ-chitosan-Linoleic acid	Drug delivery	Paclitaxel	[192]
Poly(ethylene-glycol)-*γ* carboxymethyl chitosan	Drug delivery	Dox	[193]
Chitosan/poly(ethylene glycol)– glycyrrhetinic acid	Drug delivery	Dox	[194]
Chitosan-Cholanic acid	Drug delivery	Paclitaxel	[195]
Chitosan Glyceryl monooleate	Drug delivery	Paclitaxel	[196]
Chitosan Cholesterol	Drug delivery	Epirubicin	[98]
Carboxymethyl Chitosan-PVA	wound dressings	NA	[197]
Carboxymethyl Chitosan-PVA/silk fibroin	wound dressings	NA	[198]
Carboxymethyl Chitosan-PEO	Antimicrobial	NA	[199]
Chitosan/PEO nanofibers	wound dressings	NA	[200]
Carboxymethyl Chitosan-PVA/Ag nanoparticles	antibacterial	NA	[201]
Quaternized chitosan-coPLA	antitumor	DOX	[202]
Quaternized chitosan-PVA	Antibacterial	NA	[203,204]
Quaternized chitosan-PVP	Antibacterial	NA	[205]
Quaternized chitosan-PLA	Antitumor Wound dressing	NA	[206,207]
Quaternized chitosan-Graphene	Virus removal	NA	[208]
Quaternized chitosan-Organic rectorite	Antibacterial	NA	[209]
PEG-graft chitosan	Drug release	PLGA	[210]
Poly-*ε*-caprolactone-graft chitosan	Skin tissue engineering		[211]
Iminochitosan	Wound healing	NA	[212]
Cyanoethyl chitosan	Wound dressing	NA	[213]
*N*-Methylene phosphonic chitosan	Bone grafting	NA	[214]
Hydroxypropyl Chitosan-Organic rectorite	Antibacterial	NA	[215]
Hydroxyapatite-chitosan nanocomposite	Colon cancer theraphy	Celecoxib	[216]
PGLA-chitosan	Rat glioblastoma	Carmustine (BCNU), O(6)-benzylguanine (BG) – therapeutic agents	[217]
Hyaluronic acid (HA)-CS nanoparticles	Breast cancer	miR-34a and doxorubicin (DOX)	[218]
Chitosan based glycolipid-like	Human ovarian cancer cells	Paclitaxel (PTX)	[219]
Albumin-chitosan	Mesothelioma therapy	Onconase (ONC)	[220]
Chitosan coated mixed micelles	Multidrug resistant cancer cells	siRNA and Doxorubicin	[221]
Stearic acid-grafted chitosan oligosaccharide (CSO-SA)	Cancer therapy	Polymer–drug conjugate of doxorubicin	[222]
Deoxycholic acid-O carboxymethyl chitosan	Liver cancer	Ginsenoside compound K (CK)	[223]
Cholesterol conjugated chitosan	Human lung carcinoma cells	Curcumin	[224]
Fluorescent gold nanocluster-conjugated chitosan	Lung cancer	Methotrexate	[225]
Glycol chitosan nanopolymers (psi-TGC)	knockdown of tumour Protein for cancer gene therapy	Poly siRNA	[226]
Glycol chitosan	In vivo inhibition of tumour via Gene therapy	Poly siRNA	[226]
Chitosan: poly(lactic-co-glycolic acid) nanoparticles	Silencing of aquaporin-1 cancer cells via Gene therapy	siRNA	[227]
*N*-sulfonato-N,O-carboxymethylchitosan (NOCCS)	In vivo cancer cells via Photodynamic therapy	mTHPP.	[228]

Abbreviations: PEO, poly(ethylene oxide); PVA, poly(vinyl alcohol); PEG, poly(ethylene glycol); PLGA, poly(D,L-lactide-co-glycolide); PLA, poly(L-lactide); LA, L-lactide; coPLA, poly(L-lactide-co-D,L-lactide); PVP, polyvinylpyrrolidone; PCL, poly-*ε*-caprolactone.
